# Availability and Pricing of Orthobiologic Knee Injections Vary by Practice Type but Not by Community Socioeconomic Vulnerability in Suburban New York

**DOI:** 10.1002/ars2.70074

**Published:** 2026-08-27

**Authors:** Tracy M. Tauro, Benjamin Hershfeld, Katharine Chen, Ran Lampert, Adam D. Bitterman, Randy M. Cohn, Nicholas A. Sgaglione

**Affiliations:** ^1^ Northwell Health New Hyde Park New York U.S.A.; ^2^ Department of Orthopaedic Surgery Northwell Health – Huntington Hospital Huntington New York U.S.A.; ^3^ Donald and Barbara Zucker School of Medicine at Hofstra/Northwell Hempstead New York U.S.A.; ^4^ Department of Orthopaedic Surgery Northwell Health – Long Island Jewish Medical Center Queens New York U.S.A.

## Abstract

**Purpose:**

To assess the availability, price transparency, and pricing of platelet‐rich plasma (PRP), bone marrow aspirate concentration, and stem cell injections for knee osteoarthritis (OA) among orthopaedic sports medicine (OSM) practices and alternative clinics in suburban New York and to evaluate the impact of the economic status of a population on the availability and pricing of biologic injections.

**Methods:**

Between July and September 2025, OSM practices and alternative clinics offering PRP, bone marrow aspirate concentration, or stem cell injections for knee OA were identified through American Orthopaedic Society for Sports Medicine and Arthroscopy Association of North America databases and web searches. Clinics were contacted using a standardized telephone script. Analyses used Mann‐Whitney U, Fisher's exact, and Levene's tests. Associations with socioeconomic vulnerability were evaluated using the Social Vulnerability Index and Spearman correlation.

**Results:**

Seventy‐six (50 OSM, 26 alternative) of 91 clinics offered orthobiologics. PRP availability was similar between groups (98% vs 92%; *P* = .488), whereas stem cell injections were more common at alternative clinics (38% vs 6%; *P* < .001) and bone marrow aspirate concentration was rare (0% vs 4%; *P* = .329). PRP price disclosure was similar (84% vs 85%; *P* = .99). Median PRP price was $1000 for OSM and $875 for alternative clinics (*P* = .059). Median stem cell price was $3500 at alternative clinics versus $750 at the single OSM listing (*P* = .056). Clinic location vulnerability did not differ by provider type, and PRP price was not correlated with Social Vulnerability Index.

**Conclusions:**

Orthobiologic injections for knee OA are widely available in suburban New York. PRP is nearly universal, stem cell injections remain concentrated in alternative clinics, and pricing is unrelated to community socioeconomic vulnerability.

**Clinical Relevance:**

Orthobiologic injections for knee OA are broadly available in suburban practice settings, with variable pricing and transparency that have implications for patient counseling and shared decision‐making in an out‐of‐pocket market with limited clinical evidence.

## Graphical abstract

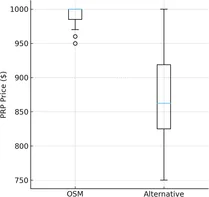

Patient preference for nonoperative management of knee osteoarthritis (OA) influences treatment decisions, because a multinational survey found that only 32% of US patients with end‐stage knee OA were willing to undergo surgery.^1^ Although nonoperative therapies such as physical therapy, nonsteroidal anti‐inflammatory drugs, and corticosteroid injections remain the standard of care, many patients now seek biologic options. Platelet‐richplasma (PRP), bone marrow aspirate concentration (BMAC), and products marketed as “stem cell” injections are being offered with the promise of symptom relief or structural benefit.

Among available orthobiologic therapies, PRP has been studied most extensively in knee OA and shows more consistent clinical benefit compared with other joints.^2^ Multiple randomized trials and meta‐analyses have shown that PRP provides clinically meaningful improvements in pain and function through short‐ and midterm follow‐up compared with placebo, corticosteroids, and hyaluronic acid, particularly in early to moderate knee OA.^3^, ^4^, ^5^

Although PRP appears to offer benefit in knee OA, the effectiveness of orthobiologic therapies in degenerative joint disease outside the knee remains inconsistent, and outcomes vary substantially by product type, preparation, and indication, because regulatory oversight is limited.^6^, ^7^, ^8^ These therapies have shown potential benefit in certain tendinopathies and soft‐tissue injuries, but broader applicability across degenerative conditions remains uncertain.^9^ Additionally, orthobiologic procedures encompass a heterogeneous group of interventions that vary substantially in composition, preparation, and adjunctive use, including combinations such as PRP administered with hyaluronic acid, which may further influence pricing.^10^ In this setting, patients encounter wide variation in product offerings, disclosure practices, and pricing transparency, with fee‐for‐service incentives that complicate shared decision‐making and raise concerns about access and financial burden.^11^

Orthobiologic therapies are rarely covered by insurance, which leaves patients responsible for substantial and widely ranging out‐of‐pocket costs.^11^, ^12^ Thus, pricing transparency can be especially important. At the same time, inconsistent regulation has resulted in broad variation in availability, marketing, and clinical practice, with some providers in both medical and holistic settings using terms such as “stem cells” and “regenerative medicine” in ways that may not accurately reflect the underlying biologic efficacy and mechanism of action of the treatments being offered.^13^ Such practices raise concerns about patient expectations, inappropriate use for financial gain, adverse events, and the risk of iatrogenic complications.

A study of clinics in the Chicago region found substantial heterogeneity in the availability, transparency, and pricing of orthobiologic injections for knee OA when comparing orthopaedic sports medicine (OSM) practices with alternative clinics.^14^ Although this provided an important early look at practice variation, the relation between these patterns and community socioeconomic context remains unclear.

The purposes of this study were to assess the availability, price transparency, and pricing of PRP, BMAC, and stem cell injections for knee OA among OSM practices and alternative clinics in suburban New York and to evaluate the impact of economic status of a population on the availability and pricing of biologic injections. We hypothesized that clinics located in higher‐income communities would be more likely to offer orthobiologic injections for knee OA at higher prices and that alternative clinics would show greater pricing variability and lower price transparency compared with OSM practices.

## METHODS

### Study Design and Eligibility Criteria

This cross‐sectional study was conducted between July and September 2025. Clinics were eligible for inclusion if they were located in suburban New York and offered at least 1 orthobiologic injection for knee OA, including PRP, BMAC, or stem cell injections. This study did not involve human subjects or protected health information and therefore did not require Insitutional Review Board (IRB) review.

### Identification of Providers

Eligible OSM practices were identified through the American Orthopaedic Society for Sports Medicine and Arthroscopy Association of North America databases, supplemented by targeted web searches. Alternative clinics were defined as nonsports medicine orthopaedic, chiropractic, pain management, or stand‐alone regenerative medicine practices advertising orthobiologic injections, consistent with prior methodology.^14^ Classification was based on publicly available practice information, including provider training, board certification, practice branding, and services advertised on practice websites and online directories. Practices staffed by fellowship‐trained OSM physicians or explicitly identifying as sports medicine practices were categorized as OSM, whereas clinics lacking sports medicine fellowship‐trained providers and marketing orthobiologic services outside a sports medicine framework were classified as alternative clinics. These OSM practices and alternative clinics were identified through Google and Yelp searches using the keywords “PRP,” “platelet‐rich plasma,” “stem cell,” “BMAC,” and “bone marrow aspirate concentrate.”

### Provider Inquiry and Data Collection

Each clinic was contacted individually by telephone. A standardized telephone script, adapted from Khan et al., was used to minimize bias and ensure consistency.^14^ Calls were conducted by 2 authors (R.L., K.C.). The script included the following: (1) “Hello, I am calling on behalf of my dad, who was looking for local places that offer knee injections for osteoarthritis. Specifically, he was looking for places that offer PRP, stem cell, or BMAC injections. Do you offer any of those services?” (2) If any of these services were offered, a follow‐up question was asked: “I know these services typically are not covered by insurance, so the cost is the biggest factor for us. Would you be able to provide an estimate for out‐of‐pocket cost for [injections offered]?” (3) If stem cell injections were available, the following was asked: “I know stem cells can come from various places. Do you know the source of the stem cells and how they are derived?” These questions referred to a single injection for 1 knee. If a receptionist could not provide the requested information, the call was transferred to an advanced practice provider when available. Each clinic was contacted up to 3 times if no response was obtained.

### Data Acquisition

For each site, data was collected on whether PRP, stem cell, or BMAC injections were offered, whether pricing information was disclosed, the quoted price of a single injection, and the reported source of stem cell products. Data was entered into Microsoft Excel (Microsoft, Redmond, WA). Providers within the same practice were grouped together to minimize duplication. If a practice had multiple offices, each office was considered a separate practice and was called individually.

### Statistical Analysis

Descriptive statistics were calculated for all continuous and categorical variables. Continuous variables, including PRP and stem cell prices, were summarized as means, standard deviations, medians, and interquartile ranges. Normality of price distributions was assessed using the Shapiro‐Wilk test, and variance equality between provider types was evaluated using Levene's test. Between‐group comparisons were performed using nonparametric methods given the non‐normal distribution of prices. Mann‐Whitney U tests were used to compare median prices between OSM and alternative clinics. Fisher's exact tests were used to compare proportions of service availability, price disclosure, and stem cell source disclosure between provider types. Associations between socioeconomic vulnerability and pricing were evaluated using Spearman correlation coefficients (price vs Centers for Disease Control Social Vulnerability Index [SVI]) and quantile (median) regression models with PRP and stem cell prices as dependent variables and SVI and provider type as independent variables. Statistical significance was set at *P *< .05. All analyses were conducted in Python (version 3.11; Python Software Foundation, Wilmington, DE).

## RESULTS

### Identification of Providers

A total of 91 clinics, including 62 OSM practices and 29 alternative clinics, were identified through initial queries. Of these, 15 were excluded, resulting in the inclusion of 76 clinics on Long Island, New York, that offered at least 1 of PRP, stem cell, and/or BMAC injections as an in‐clinic procedure for the management of knee OA. The final cohort comprised 50 OSM practices and 26 alternative clinics. Geographically, 37 clinics were located in Nassau County and 39 in Suffolk County.

### Product Availability

Among 76 clinics offering in‐clinic orthobiologic injections for knee OA, there was no significant difference between OSM and alternative clinics in the availability of PRP injections (98% vs 92%; *P* = .488). Stem cell injections were offered significantly more often at alternative clinics compared with OSM practices (38% vs 6%; *P* < .001). BMAC injections were not offered by any OSM clinic and were reported by only 1 alternative clinic (0% vs 4%; *P* = .329) (Table 1).

**TABLE 1:** Availability and Transparency of Orthobiologics by Provider Type

	Orthopaedic Sports Medicine (n = 50)	Alternative Clinics (n = 26)	*P* Value
Offer PRP injections∗	49:1 (98)	24:2 (92)	.488
PRP injection price disclosed∗	42:8 (84)	22:4 (85)	≥.99
Offer stem cell injections∗	3:47 (6)	10:16 (38)	**<.001**
Stem cell injection price disclosed∗	1:49 (2)	7:19 (27)	.569
Stem cell source disclosed∗	1:49 (2)	5:21 (19)	**.016**
Offer BMAC injections∗	0:50 (0)	1:25 (4)	.329
BMAC injection price disclosed∗	0:50 (0)	0:26 (0)	‐

*Note*: Data are presented as n (%) unless otherwise indicated. Bold or marked *P* values denote statistical significance (*P* < .05).

BMAC, bone marrow aspirate concentrate; PRP, platelet‐rich plasma.

∗ Denotes a dichotomous categorical variable presented as yes/no.

### Price Transparency

There was no difference between provider types in the likelihood of disclosing PRP prices (84% vs 85%; *P* = .99). Stem cell prices were less frequently disclosed overall but did not differ significantly between groups (33% vs 70%; *P* = .569). Alternative clinics were significantly more likely to disclose the biological source of their stem cell products (19% vs 2%; *P = *.016). No clinics reported BMAC pricing.

### Price Comparison

**FIGURE 1:**
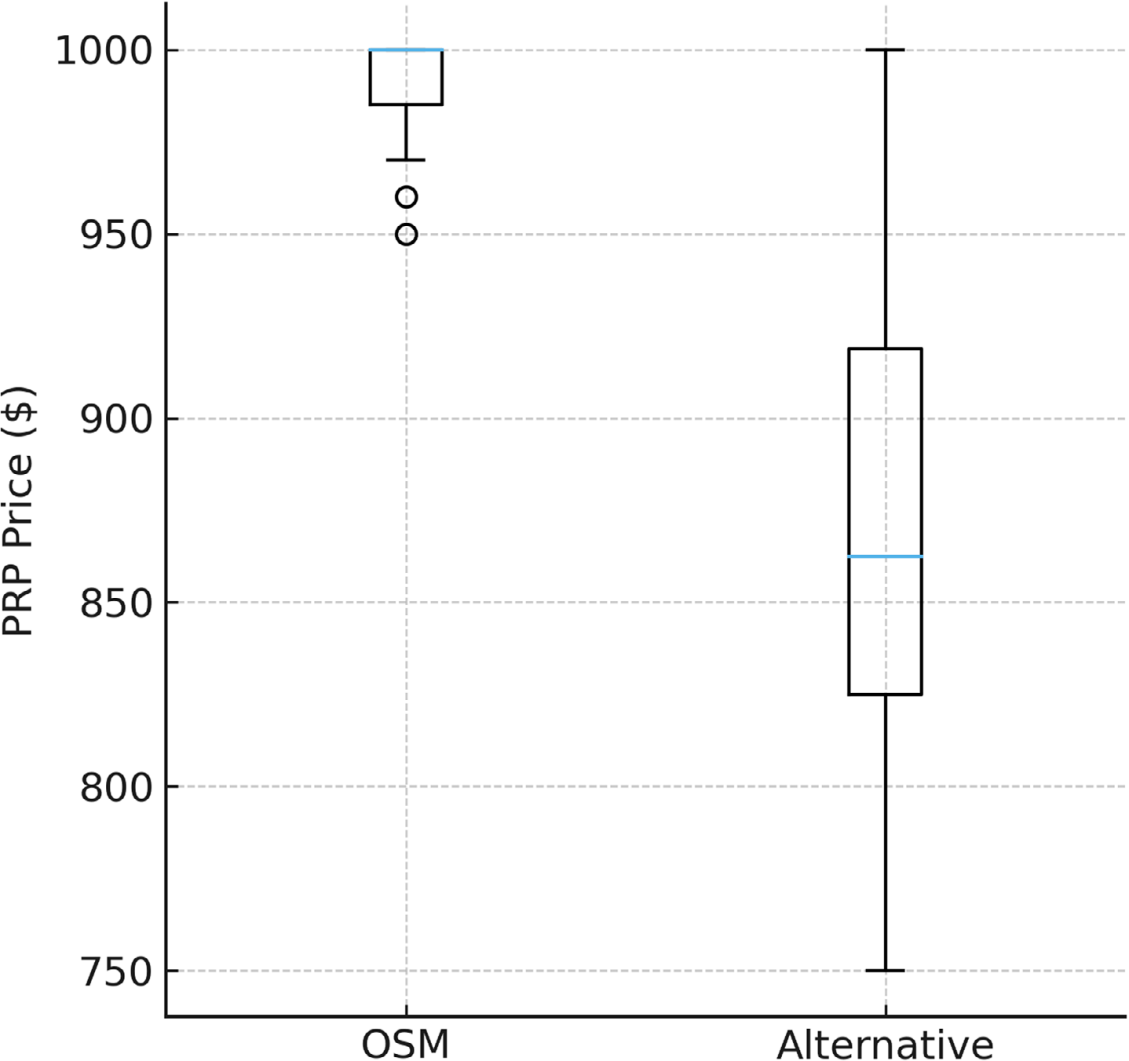
Box plot showing the distribution of PRP injection prices by provider type, showing overlapping ranges and a nonsignificant trend toward higher median pricing among OSM practices. (OSM, orthopaedic sports medicine; PRP, platelet‐rich plasma.)

Among clinics providing price information, the median PRP price was $1000 (interquartile range, $800‐$1000) overall, with OSM practices averaging $992 ± $243 and alternative clinics averaging $823 ± $185. This difference trended toward significance but did not reach statistical significance (*U* = 434, *P* = .059). Variability in PRP pricing was similar across provider types (Levene's *P* = .476), and Shapiro‐Wilk testing confirmed a non‐normal distribution (*P* < .001 for both groups) (Figure 1).

Stem cell pricing was available for 1 OSM clinic ($750) and 7 alternative clinics (median, $3500; mean, $3779 ± $2246). Although the descriptive difference was large, the comparison did not reach statistical significance (*P = *.056) because of the small number of OSM listings (Table 2).

**TABLE 2:** Pricing of PRP and Stem Cell Injections by Provider Type

	Orthopaedic Sports Medicine	Alternative Clinics	*P* Value
PRP injection price, mean (SD), $	992 (243)	823 (185)	‐
PRP injection price, median [IQR], $	1000 [985‐1000]	875 [750‐1000]	‐
Mann‐Whitney U test	*U* = 434		.059
Stem cell injection price, mean (SD), $	750 (NA)	3779 (2246)	‐
Stem cell injection price, median [IQR], $	750 [750‐750]	3500 [2750‐4750]	‐
Mann‐Whitney U test	*U* = 2		.056

*Note*: Data are presented as mean (SD) or median [IQR] as appropriate.

IQR, interquartile range; PRP, platelet‐rich plasma; SD, standard deviation.

### Socioeconomic Analysis

Clinic locations were further analyzed using the Centers for Disease Control SVI to assess the socioeconomic context of practice locations. The mean SVI across all clinics was 0.18, indicating generally low overall regional vulnerability. OSM practices were located in areas with slightly lower vulnerability (mean SVI ≈ 0.16) compared with alternative clinics (mean SVI ≈ 0.22), though this difference was not statistically significant (*P* = .31).

**FIGURE 2:**
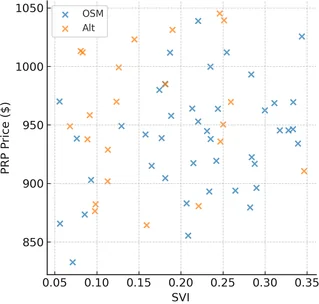
Scatterplot showing the relation between PRP injection price and SVI score, indicating no meaningful correlation between clinic pricing and community‐level socioeconomic vulnerability. (OSM, orthopaedic sports medicine; PRP, platelet‐rich plasma; SVI, Social Vulnerability Index.)

A Spearman correlation between PRP price and SVI showed a weak, positive, and nonsignificant association (*ρ* = 0.18, *P* = .154), indicating that clinic pricing did not meaningfully vary by neighborhood socioeconomic conditions (Figure 2).

### Regression Analyses

Quantile regression modeling of PRP pricing (n = 64) by provider type and SVI revealed that OSM practices listed median PRP prices approximately $100 higher than alternative clinics. Although statistically significant because of large sample precision, the effect was economically negligible (+$0.40 per 1.0‐unit increase in SVI). For stem cell pricing, neither provider type (*P* = .475) nor SVI (*P* = .766) significantly predicted cost.

## DISCUSSION

Orthobiologic injections for knee OA were widely available across suburban practice settings, with PRP offered by nearly all providers, stem cell therapies concentrated in alternative clinics, and BMAC rarely available. Pricing was not meaningfully associated with community socioeconomic vulnerability. Price disclosure for PRP was high across both groups, whereas stem cell pricing and source disclosure were more common among alternative clinics.

Contrary to initial expectations, OSM practices did not charge meaningfully higher PRP prices compared with alternative clinics, and PRP cost was not associated with local socioeconomic vulnerability. Regression modeling confirmed that provider type modestly influenced listed pricing, but SVI had no economically meaningful effect, suggesting that orthobiologic pricing on Long Island is driven primarily by practice‐level characteristics rather than community socioeconomic conditions. Together, these results indicate that although biologic therapies are widely available, pricing and transparency remain inconsistent, and local economic context does not appear to influence market behavior within this specific suburban region.

These findings align with prior reports documenting the widespread availability of PRP and the more frequent offering of stem cell injections by alternative clinics. Khan et al. found that OSM practices and alternative clinics offered PRP at similar rates, whereas alternative clinics were significantly more likely to advertise stem cell injections.^14^ Prior regional and national surveys have shown steady expansion of PRP use among OSM practices, with reported offering rates ranging from 36% in Miami‐Dade County in 2019 to over 70% nationally in 2020.^11^, ^12^ In this study, which specifically examined clinics already providing orthobiologic injections, PRP availability exceeded 90% across both provider types, consistent with the broader trend toward increasing adoption of PRP therapies over time.

For stem cell injections, the 6% prevalence among suburban New York OSM practices is lower than the 21% to 25% prevalence reported in prior national and regional surveys.^11^, ^12^ Conversely, 38% of alternative clinics in this cohort offered stem cell injections—lower than the 70% reported in Khan et al.'s analysis, yet still substantially higher than among OSM practices.^14^ This disparity reinforces that stem cell injections remain concentrated in the alternative clinic marketplace. BMAC injections were rarely offered, consistent with prior observations that BMAC availability is limited and pricing data are sparse. This likely reflects the complexity and procedural feasibility, because PRP requires only a peripheral blood draw that can be performed in most office settings, whereas BMAC harvesting involves bone marrow aspiration, which is more invasive and time‐intensive and requires specialized equipment and training.

Pricing patterns also differed across studies. PRP pricing among suburban New York OSM practices (median $1000) was broadly comparable to values previously reported in Miami ($897) and the Midwest ($703).^11^, ^12^ Although OSM clinics in this cohort listed higher PRP prices than alternative clinics, this difference was not statistically significant, contrasting earlier findings that showed clear cost disparities. Stem cell injections remained substantially more expensive than PRP, with median prices of roughly $3500 among alternative clinics compared with $750 among OSM practices (based on a single disclosed value), consistent with prior reports of wide pricing variability and higher costs in alternative markets.

Currently, most orthobiologic injections for knee OA remain out‐of‐pocket expenses, because insurance coverage for BMAC and stem cell injections is uncommon and PRP coverage remains limited. In this setting, pricing transparency assumes greater importance for patient decision‐making. In the present study, PRP prices were modestly higher at OSM practices compared with alternative clinics, although this difference did not reach statistical significance. OSM practices showed narrower PRP price distributions, whereas alternative clinics exhibited greater pricing variability, particularly for stem cell injections. Similar patterns of pricing heterogeneity across provider types have been reported previously, suggesting that variability is a recurring feature of the orthobiologics marketplace.^14^

The greater availability of stem cell injections in alternative clinics raises important regulatory and safety concerns. The US Food and Drug Administration has discouraged the use of many products marketed as stem cell therapies for musculoskeletal applications, and regulatory oversight varies based on product source and degree of manipulation.^15^, ^16^ Despite these constraints, such therapies continue to be marketed in clinical practice, often with limited clarity regarding biologic composition or mechanism of action.^17^

By incorporating a socioeconomic framework using the Centers for Disease Control SVI, this study shows that the availability and pricing of orthobiologic injections appear largely independent of community‐level vulnerability, suggesting that market behavior is driven primarily by practice‐level factors rather than local economic conditions. These findings show the need for greater transparency and standardization in an out‐of‐pocket marketplace where clinical evidence remains inconclusive and pricing highly variable, and they highlight the continued need for rigorous evidence‐based outcome studies. As patient demand and provider marketing continue to expand, enthusiasm for orthobiologic injections may outpace the current scientific evidence, and these therapies remain insufficiently validated for widespread clinical adoption.^18^

### Limitations

This study has several limitations. The relatively low number of clinics offering certain orthobiologic interventions, particularly BMAC and stem cell injections, may have limited statistical power to detect true differences between provider types, introducing the potential for type II error. As a result, nonsignificant findings related to these outcomes should be interpreted with caution. Group sizes were unequal between OSM practices and alternative clinics, which may further limit power for some between‐group comparisons. Orthobiologic offerings were classified based on information provided during telephone inquiries, and variation in terminology, preparation, and adjunctive use of biologic products may introduce misclassification or imprecision. Additionally, quoted prices may differ from those ultimately billed. Nonresponse may have biased the results if more transparent practices were more likely to disclose. For stem cell pricing among OSM practices, only a single data point was available, limiting comparison. This analysis was confined to Nassau and Suffolk counties on Long Island, New York, which may not generalize to other US markets. Finally, the cross‐sectional design cannot assess how pricing or availability may change over time.

## CONCLUSIONS

Orthobiologic injections for knee OA are widely available in suburban New York. PRP is nearly universal, stem cell injections remain concentrated in alternative clinics, and pricing is unrelated to community socioeconomic vulnerability.

## DISCLOSURES

The author (N.A.S.) declares the following financial interests/personal relationships which may be considered as potential competing interests: N.A.S. reports consulting fees from Biomet and Embody (consulting/advisory); leadership or fiduciary role in other board, society, committee, or advocacy group, paid or unpaid, in the Arthroscopy Association of North America and Sports Medicine and Arthroscopy Review (board membership); stock or stock options in Biomet, Wolters Kluwer Health, and Zimmer (equity or stock ownership); receipt of equipment, materials, drugs, medical writing, gifts, or other services from ReGen Biologics and Zimmer (nonfinancial support); other financial or nonfinancial interests from Wolters Kluwer Health (funding grants). The other authors (T.M.T., B.H., K.C., R.L., A.D.B., R.M.C.) declare that they have no known competing financial interests or personal relationships that could have appeared to influence the work reported in this article.
